# National Survey of Workplaces Handling and Manufacturing Nanomaterials, Exposure to and Health Effects of Nanomaterials, and Evaluation of Nanomaterial Safety Data Sheets

**DOI:** 10.1155/2016/8389129

**Published:** 2016-07-31

**Authors:** Jeongho Kim, Il Je Yu

**Affiliations:** ^1^Chungju District Office of Ministry of Employment and Labor, 3-3 kugwon-daero, Chungju 27428, Republic of Korea; ^2^Institute of Nanoproduct Safety Research, Hoseo University, 165 Sechul-ri, Baebang-eup, Asan 31499, Republic of Korea

## Abstract

A national survey on workplace environment nanomaterial handling and manufacturing was conducted in 2014. Workplaces relevant to nanomaterials were in the order of TiO_2_ (91), SiO_2_ (88), carbon black (84), Ag (35), Al_2_O_3_ (35), ZnO (34), Pb (33), and CeO_2_ (31). The survey results indicated that the number of workplaces handling or manufacturing nanomaterials was 340 (0.27% of total 126,846) workplaces. The number of nanomaterials used and products was 546 (1.60 per company) and 583 (1.71 per company), respectively. For most workplaces, the results on exposure to hazardous particulate materials, including nanomaterials, were below current OELs, yet a few workplaces were above the action level. As regards the health status of workers, 9 workers were diagnosed with a suspected respiratory occupational disease, where 7 were recommended for regular follow-up health monitoring. 125 safety data sheets (SDSs) were collected from the nanomaterial-relevant workplaces and evaluated for their completeness and reliability. Only 4 CNT SDSs (3.2%) included the term nanomaterial, while most nanomaterial SDSs were not regularly updated and lacked hazard information. When taken together, the current analysis provides valuable national-level information on the exposure and health status of workers that can guide the next policy steps for nanomaterial management in the workplace.

## 1. Introduction 

With the increasing application of nanotechnology in new areas of industrial convergence, there is also increasing concern over the health effects on workers primarily exposed to nanomaterials and on consumers. As a result, many countries are now investigating the populations exposed to nanomaterials, use of nanomaterials, and status of products containing nanomaterials; also information-gathering legislation has already been adopted in several countries. In 2012, France introduced mandatory reporting for the production, distribution, or importation of more than 100 g of nanomaterials per year [1]. In Canada, Environment Canada and Health Canada proposed a new approach to address certain nanomaterials under the Canadian Environmental Protection Act, 1999; a consultation document on a Proposed Approach to Address Nanoscale Forms of Substances on the Domestic Substances List was published with a public comment period ending on May 17, 2015, and a domestic substance list relevant to nanomaterials was also published [2]. Moreover, the US EPA proposed one-time reporting and recordkeeping requirements for new and existing chemical substances that are nanoscale materials under the authority of Section 8(a) of the TSCA, where persons who manufacture or process these chemical substances as nanoscale materials are required to notify the EPA of certain information, including the specific chemical identity, production volume, methods of manufacture and processing, use, exposure, and release information, and available health and safety data [3]. Meanwhile, several countries have conducted surveys on the use and production of nanomaterials. In 2005, the Federal Institute for Occupational Health and Safety (BAuA) in Germany carried out a questionnaire on activities involving nanomaterials and found that 21% (*n* = 45) of companies perform activities involving nanomaterials [4]. In a second survey by BAuA in 2011 on the handling and use of nanomaterials, which included more than 1,700 companies and institutions, it was found that SiO_2_, TiO_2_, polymers (synthesis), and carbon black were the main nanomaterials used by industry [5]. In Denmark, a questionnaire was conducted in 2010 to ask if companies anticipated producing, importing, or using nanomaterials, and 27 out of 162 responded positively [6]. In a Swiss survey in 2010, 309 workers were reported to have been potentially exposed to nanoparticles in 586 companies [7], while a French national survey in 2010 to identify the types of nanomaterials in the French market reported that 288 workers had been potentially exposed to nanoparticles [8]. Plus, a total of 61 companies manufacturing engineered carbon nanotubes (ECN) were identified in the United States in 2011 [9].

Despite published results on exposure to nanomaterials, there has been no national-level data analysis of exposure to nanomaterials. Furthermore, there have been relatively few studies on work-related symptoms and diseases among workers handling engineered nanomaterials. In a Taiwanese study, the only significantly work-related symptom was found to be sneezing, a work-related dry cough and productive cough were found to be more prevalent in workers handling nanomaterials than in the controls, and the only disease found to be significantly worsened by work was allergic dermatitis [10]. The OECD conducted an informal survey that compiled information regarding methods and models for assessing exposure to manufactured nanomaterials and found that airborne particulate and the respiratory track were the most easily exposed type of nanomaterial and pathway, respectively [11]. Although the Korean Occupational Safety & Health Act does not specifically regulate nanomaterials in the workplace environment; exposure to hazardous materials, regardless of size, must be measured annually and the exposure results reported to the Ministry of Employment and Labor and registered in the National Database. In addition, the health status of workers manufacturing and handling hazardous materials, regardless of size, is required to be monitored annually and the results are required to be reported to the Ministry of Employment and Labor and registered in the National Database. While such data are not directly relevant to nanomaterial exposure and health outcomes, they can still provide a larger picture of the status of nanomaterial exposure and the health status of workers manufacturing and handling nanomaterials.

European Agency of Safety and Health at Work (EU OSHA) published a report on risk perception and risk communication with regard to nanomaterials in the workplace in 2007 [12]. In this report, quality of 30 SDSs for nanomaterials was examined in 2007 in Nordic countries whether the toxicological information was reasonable and accurate [13]. The conclusions drawn were that there was a shortage of data to inform the SDS: there were not sufficient published toxicological and physical characterization data on the nanomaterials in the scientific literature. There was also a lack of data on whether the properties of the nanomaterials were different from those composed of larger particles. In addition, the instructions in the EC Directive 2001/58/EC were not appropriate for the risk assessment of nanoparticles. Every five years, Korea conducts a national workplace environment survey according to the Industrial Safety and Health Act. The survey does not include sampling of workplace; however, it covers all workplaces in Korea. A questionnaire is sent to every workplace and trained interviewers visit assigned workplaces and confirm the questionnaire responses prepared by the business owners. The national workplace environment survey conducted in 2014 included several additional items regarding information on nanomaterials. Safety data sheets (SDSs) on nanomaterials were also requested from manufacturers.

Accordingly, this paper presents the results of the 2014 Korean workplace environment survey as relevant to nanomaterials, preliminary data on the exposure and health status of workers manufacturing and handling nanomaterials, and SDS information on nanomaterials.

## 2. Materials and Methods

### 2.1. National Workplace Environment Survey

In 2014, the Korean Ministry of Employment and Labor (MOEL) conducted a national survey on the handling and manufacturing of nanomaterials. This was the first survey on the use or manufacture of nanomaterials for the Korean manufacturing industry. The survey was carried out by the Korea Occupational Safety and Health Agency (KOSHA), an agency under MOEL. Sampling for the number of companies to be surveyed was designed 150,000 (41.7%) of the total 359,745 companies as a population, including all 120,195 companies with 5 or more workers, all 13,179 companies of 11 industries and 6,626 (3.9%) sample companies with fewer than 5 workers, and 10,000 (17.4%) sample companies with 5 or more workers in nonmanufacturing industry. As a result, the number of companies included in the survey was 126,846 (based on the results of the national workplace environment survey, Korea Occupational Safety and Health Agency, 2014), consisting of 100,773 manufacturing companies with 5 or more workers (number of manufacturing companies with 5 or more workers and industrial accident compensation insurance on January 1, 2014, and excluding companies that had closed, were self-employed, or were bankrupt from the total number of 120,195), 16,073 sample manufacturing companies among 182,110 with fewer than 5 workers, and 10,000 sample companies from the nonmanufacturing sector (Table 1). The amount of nanomaterials used in the workplace was not surveyed in this survey since the amount of nanomaterial has been surveyed by the Ministry of Environment. Total amount of nanomaterials used in 2011 in industry was 8,704 ton per year. The data on the amount used by nanomaterial in 2011 are available in Supplement Table 1 (see Supplementary Material available online at http://dx.doi.org/10.1155/2016/8389129).

#### 2.1.1. Survey Methods

KOSHA carried out the survey in collaboration with other organizations relevant to occupational health and safety and recruited field surveyors with a national certificate in occupational safety and health or graduating/graduated students majoring in science or engineering. KOSHA provided on-the-job training to all the field surveyors. After receiving the initial questionnaire, each company received a follow-up visit by a field surveyor to confirm the answers provided by the company employee in charge of health, safety, and environment. Furthermore, the number of companies identified as handling or manufacturing nanomaterials was added to the results of the national survey.

#### 2.1.2. Survey Contents

The contents of the questionnaire used in the national survey were mainly focused on the handling and manufacturing of chemicals, including nanomaterials, as well as the use of the hazardous equipment and facilities. The headings related to nanomaterials were as follows:

- General information on the company, including type of industry and number of workers.
- Total number of nanomaterials and nanoproducts handled or manufactured by the company.
- Type of handling or manufacturing, such as synthesis, processing, or production.

In the questionnaire, “handling and manufacturing” means the synthesis, processing, or production of nanomaterials, “synthesis” means to synthesize nanomaterials from raw nonnanomaterials, “processing” means to manufacture nanomaterials by coating, surface treatment, or crushing to a smaller size, and “production” means to make a final product using raw materials or nanomaterials or the addition of additives. The contents of the national survey questionnaire are presented as follows.

*Contents of Questionnaire Used in National Survey*

Do you handle or manufacture nanomaterials?

1. yes
2. no

Way of handling or manufacturing nanomaterials

1. synthesis
2. processing
3. production

Nanomaterials handled or manufactured

1. C^60^
2. Carbon black
3. Ag
4. Fe
5. Au
6. Pb
7. La
8. TiN
9. TiO_2_
10. CeO_2_
11. ZnO
12. SiO_2_
13. Al_2_O_3_
14. Sb_2_O_5_
15. SnO_2_
16. CoO
17. Nanoclay
18. Polymers
19. Nano-polystyrene
20. Dendrimers
21. carbon nanotubes or carbon nanofibers
22. Other ( )

### 2.2. Workplace Measurements and Health Examination of Workers

To evaluate worker exposure to nanomaterials and the health effects on workers potentially exposed to nanomaterials, the 2014 results for the workplace air measurements, regardless of size, and special health examinations of workers were collected and analyzed. The results were collected from employers manufacturing or handling listed hazardous materials, regardless of size (including nanoscale materials), according to the Occupational Safety and Health (OSH) Act. Most of the workplace air measurements and worker health examination results were from the same workplaces that submitted MSDSs for this study. The air filter samples were collected and analyzed using the work environment measurement methods described in the OSH Act (notification). The air samplings were conducted by MOEL-designated occupational health service institutes specialized in measuring the work environment. Nanoscale characteristics were not considered for the workplace air measurements since there is no current obligation to measure nanomaterials. The special health examinations of workers potentially exposed to hazardous materials were conducted by MOEL-designated health institutes (hospitals, etc.) with occupational health services. An employer shall conduct a special health examination for workers to protect workers' health, and if it is deemed necessary for maintaining the health of workers as a result of the health examination, the employer shall change the work of the workers, shorten the working hours, improve facilities, or take other proper measures according to the OSH Act.

### 2.3. Collection and Evaluation of Nanomaterial SDSs

The SDSs for the nanomaterials/nanoproducts prepared or kept by the companies were sorted by language and country of origin as follows:

- SDS prepared in Korean as domestic product.
- SDS prepared in Korean as domestic product (provided by KOSHA).
- SDS prepared in Korean as imported product.
- SDS prepared in English as domestic product.
- SDS prepared in English as imported product.

To evaluate the nanomaterial SDS information, the contents were compared with the ISO TR 13339 [14] Nanomaterials: Preparation of Material Safety Data Sheet (MSDS). If the SDS content did not include all the headings in the ISO guideline, the SDS was deemed unsatisfactory as regards the reliability standard for the respective heading. In addition, the SDSs were checked against the Korean standard for the classification, labeling, and SDS of chemicals, which implements the UN-GHS [15] and takes the form of notification as stated in the Occupational Safety and Health Act of MOEL. The nanomaterial SDS contents were evaluated based on ISO TR 13329 as described in the following:

- SDS prepared by one or more competent persons.
- Recommended use.
- Format of SDS.
- No blanks under any heading (plus, whether headings were same as UN-GHS format).
- Date SDS was prepared.
- Identity of organization that prepared SDS.
- Revision date (review period).
- All hazards associated with manufactured nanomaterials of Ag, TiO_2_, CNTs, SiO_2_, carbon black, CeO_2_, or mixture including those materials.
- Occupational exposure limit for CNTs and TiO_2_.

#### 2.3.1. SDS Preparation by One or More Competent Persons (Based on ISO TR 13329 Section 4.1.2)

The competency of the person(s) preparing the SDS was evaluated based on the inclusion of the name of the person(s) or department that prepared the SDSs. Thus, if the SDS included the name of the person(s) or department, for example, production department, HSE department, product safety department, or company translated SDS with a contact address, such as an e-mail address, it was regarded as prepared according to the ISO guideline. However, if the SDS only included the name of the supplier, it was not regarded as correctly prepared according to the ISO guideline. Plus, if the SDS only included the name of the supplier in the export country or just the logo of the company or supplier, it was not regarded as correctly prepared according to the ISO guideline, as the supplier or SDS preparer differed from the manufacturer.

#### 2.3.2. Recommended Use of Product Included in SDS (Based on ISO TR 13329 Section 4.1.2)

When the recommended use of the product was included in the SDS, the SDS was regarded as prepared according to the ISO guideline.

#### 2.3.3. SDS Format (Based on ISO TR 13329 Section 4.1.3)

The format of each SDS was checked as to whether it differed from the GHS format, such as missing headings, the arrangement, and phrasing.

#### 2.3.4. Blanks under Headings (Based on ISO TR 13329 Section 4.1.4)

Each of the 16 SDS headings was checked as to whether it included the required information or such phrases as “not available” or “no data” in the case that relevant information could not be found. The headings were also evaluated as to whether available information was included. Thus, if information was missing under the headings for physiochemical properties or physical and chemical properties and toxicological information, the SDS was regarded as not prepared according to ISO TR 13329.

#### 2.3.5. Review Period

Although ISO TR 13329 does not include any definitive statement on the review period for an SDS, the rapidly changing state of knowledge in the areas of nanotechnology and nanosafety means that an SDS should be reviewed periodically to keep it up to date. Thus, whenever new or significant information on the hazards of a particular material becomes available, this information should be included in the SDS.

#### 2.3.6. Hazards Associated with Manufactured Nanomaterials (Based on ISO TR 13329 Section 4.2.2)

For information on the health hazards associated with nanomaterials or their mixtures, the SDSs were compared for six representative nanomaterials, which have been reported to cause health effects in* in vitro* or* in vivo* animal studies [16–21]. Nanomaterial health hazard data from the OECD WPMN (working party on manufactured nanomaterials) safety sponsorship programme and dossiers on nanomaterials have already been published and are publicly available as follows.

(i) Nanomaterials: inflammation of respiratory tract, cardiovascular and other extrapulmonary effects, and pulmonary and systemic effects of inhaled nanoparticles.
(ii) Silver (Ag): argyria, respiratory sensitizer subcategory 1B and specific target organ toxicity-repeated exposure category 1.
(iii) Titanium dioxide (TiO_2_): possibly carcinogenic to humans Group 2B according to IARC monograph.
 “There is some evidence that breathing in large quantities of TiO_2_ nanoparticles over a long period of time may cause toxic effects due to impairment of the normal lung clearance mechanisms”.
(iv) Carbon nanotubes (CNTs), including multiwalled carbon nanotubes (MWCNTs): possibly carcinogenic to humans as MWCNT-7 (no company handling or manufacturing MWCNT-7 was included in this study), specific target organ toxicity-repeated exposure category 1.
(v) Silica dioxide (SiO_2_): specific target organ toxicity-repeated exposure category 1.
 “Cellular uptake, size- and dose-dependent cytotoxicity, increased reactive oxygen species levels and pro-inflammatory stimulation, and largely reversible lung inflammation, granuloma formation and focal emphysema, with no progressive lung fibrosis”.
(vi) Carbon black: possibly carcinogenic to humans 2B.
(vii) Cerium dioxide (CeO_2_): specific target organ toxicity-repeated exposure category 1.

Therefore, if the relevant hazard information was stated under heading 2 “Hazard Identification” or heading 11 “Toxicological Information”, the SDS was regarded as prepared according to ISO TR 13329.

#### 2.3.7. Occupational Exposure Limits (Based on ISO TR 13329 Section 4.2.8.2)

In the case of nanomaterials, the two relevant occupational exposure limits (OELs) are for CNTs and TiO_2_. The US NISOH has already recommended OELs for carbon nanotubes and fibers and ultrafine TiO_2_. Thus, the SDSs were checked for the inclusion of OELs for CNTs or TiO_2_.

## 3. Results

### 3.1. Results of National Survey

#### 3.1.1. Overview of Companies Handling or Manufacturing Nanomaterials in Korea

The total number of manufacturing workplaces that participated in the national workplace environment survey in 2014 was 126,846. This study mainly included the workplaces hiring 5 or more workers with Occupational Accidents Compensation Insurance (OACI) on January 1, 2014, numbering 100,773, the workplaces hiring fewer than 5 workers, numbering 16,073, and a sample of nonmanufacturing workplaces, numbering 10,000 (Table 1).

The total number of workplaces handling or manufacturing nanomaterials was 340 (0.27%): 75 (22.1%) for synthesis, 121 (35.5%) for processing, 75 (22.1%) for production, and 69 (20.3%) for other (Table 1). “Other (*n* = 69)” was interpreted as workplaces involved in two or more operations and workplaces using nanomaterials or already checked in the manufacturing or handling that implies meaning of synthesis, processing, or production. The number of nanomaterials handled or manufactured by the workplaces was 546, while the number of products containing nanomaterials was 583. Thus, the average number of nanomaterials per company was 1.60 and the average number of nanoproducts per company was 1.71 (Table 1). 45,750 workers from 340 workplaces are estimated for potentially associated with exposure to manufactured nanomaterials. The number of nanomaterials handled or manufactured or manufactured for products was 583, as shown in Table 2.

### 3.2. Results of Exposure to Nanomaterials and Health Examinations

#### 3.2.1. Results of Workplace Exposure to Nanomaterials

Among the 136 workplaces that implemented workplace air sampling according to the Occupational Safety and Health Act, 63 (46.3%) workplaces reported hazardous particulate material detected or nondetected. Notwithstanding, most of the workplace air measurement data indicated that the particulate material exposure was under the current occupational exposure limits (Table 3).

#### 3.2.2. Results of Special Health Examinations of Workers

A total of 48 (70.6%) companies implemented special health examinations for 1,230 workers handling and manufacturing nanomaterials, including 385 workers related to SiO_2_, 44 to ZnO, and 801 to other hazardous materials (Table 4). As a result of the health examinations, 9 cases of suspected occupational disease were diagnosed by occupational physicians, where 2 cases were related to SiO_2_, 2 cases to ZnO, and 5 cases to other hazardous materials. Regular follow-up health examinations were required for 7 workers, including 5 related to SiO_2_, 1 to ZnO, and 1 to other hazardous materials (Table 4). These 9 cases were diagnosed by occupational medicine physicians under the Occupational Safety and Health Act in Korea. The suspected occupational disease cases mean that the workers were at the status of disease or able to progress to the status of disease.

### 3.3. Evaluation of SDS Information on Nanomaterials or Nanoproducts (Based on ISO TR 13329)

125 SDSs were collected from 31 nanomaterial-relevant manufacturing workplaces and their completeness and reliability were evaluated. 56 of the SDSs were from companies manufacturing electronic components or semiconductor devices, 17 were from companies manufacturing organic chemicals, and 13 were from companies manufacturing rubber products, as described in Table 5. “Other” included companies manufacturing metal products (1 workplace with 6 SDSs), chemical products (1 workplace with 1 SDS), wholesale and retail trade and customer appliance maintenance and repair (1 workplace with 2 SDSs), publishing activities (1 workplace with 3 SDSs), and soil and stone products (1 workplace with 11 SDSs). And the SDSs were collected by nanomaterial as described in Table 6. The 125 SDSs were evaluated as regards their completeness and reliability, as described in Table 7. According to the Korean Occupational Safety Health Act, an SDS should be written or translated into Korean. Among the 125 SDSs, 15 were not written in Korean, as shown in Table 7.

#### 3.3.1. Competent Person and Recommended Use (Based on ISO TR 13329 Section 4.1.2)

ISO TR 13329 Section 4.1.2 states the following: “the SDS should be prepared by one or more competent persons who should take into account the specific needs of the intended audience, as far as they are known.” The number of SDSs that included the name of a competent person(s) was 19 (15.2%), while the number that included the recommended use(s) of the nanomaterial was 98 (78.4%) (Table 7).

#### 3.3.2. Format of SDS (Based on ISO TR 13329 Section 4.1.3)

In Korea, the SDS format is regulated in the notification called “standard on classification, labeling and SDS of chemicals (based on GHS)” pursuant to the Occupational Safety and Health Act. Therefore, an SDS should be prepared according to the notification (the Act). Among the 125 collected SDSs, 111 (88.8%) were prepared according to the notification (the Act) (Table 7).

#### 3.3.3. Relevant Information (Based on ISO TR 13329 Section 4.1.4)

ISO TR 13329 Section 4.1.4 states the following: “if relevant information for any of the required 16 SDS sections cannot be found, this fact should be indicated on the SDS in the appropriate section using phrases such as ‘not available'. The SDS should have no blanks under any of the headings.” In the present evaluation, the number of SDSs with no blanks under any heading was 108 (86.4%) (Table 7).

#### 3.3.4. Periodic Review and Reissue

An SDS must be reviewed periodically in order to keep it up to date with new or significant information on the hazards of certain materials. In Canada, an SDS must be reviewed and reissued at least every 3 years [22]. Among the 125 collected SDSs, 51 (40.8%) had been reviewed and reissued within 3 years, 48 (38.4%) had not been reviewed or reissued for more than 3 years, and 26 (20.8%) were still within 3 years of first being issued (Table 7).

#### 3.3.5. Hazard Identification (Based on ISO TR 13329 Section 4.2.2)

The inclusion of health hazard information for 6 representative nanomaterials (Ag, TiO_2_, CNTs, SiO_2_, carbon black, and CeO_2_) was checked in the 85 SDSs that included these nanomaterials. Among the 85 SDSs, 21 (24.7%) included health hazard information, whereas 64 (75.3%) did not (Table 7).

#### 3.3.6. Hazard Identification: Health Hazard Information (Based on ISO TR 13329 Section 4.2.2)

ISO TR 13329 states that “the SDS should describe all of the hazards associated with the manufactured nanomaterial or mixture for which the SDS is being prepared.” Plus, if the manufactured nanomaterial or mixture is classified according to GHS, the specific hazard and category should be identified. As presented in Table 7, only 15 (17.6%) SDSs included specific health hazard information on the 6 representative nanomaterials: 7 TiO_2_ (53.8%), 3 Ag (50.0%), 3 CNTs (60.0%), 1 CeO_2_ (3.8%), and 1 carbon black (6.7%).

#### 3.3.7. Statement of Manufactured Nanomaterial (Based on ISO TR 13329 Section 4.2.3.1)

ISO TR 13329 Section 4.2.3.1 states that “if a nanomaterial has the same Chemical Abstracts Service (CAS) number as the bulk (non-nanoscale) material, use that CAS number, but also state that the material is a manufactured nanomaterial according to ISO's definition or other applicable definitions.” When checking the inclusion of a statement of manufactured nanomaterial under heading 3 (composition/information on ingredients), only 4 (3.2%) SDSs included this statement (for CNTs) related to the composition or product name (Table 7).

#### 3.3.8. Accidental Release Measures (Based on ISO TR 13329 Section 4.2.6.1)

The number of SDSs that included a cleanup method for spills, leaks, or releases was 3 (2.4%), while 4 (3.2%) included a cleanup method for dry manufactured nanomaterials and 3 (2.4%) included a wet-wiping method for spills of liquids containing manufactured nanomaterials (Table 7).

#### 3.3.9. Occupational Exposure Limit Values (Based on ISO TR 13329 Section 4.2.8.2)

The US NIOSH recommended that occupational exposure to carbon nanotubes (CNTs) and carbon nanofibers (CNFs) be controlled to reduce the potential risk of certain work-related lung effects. Thus, as a recommended exposure limit, employers should reduce worker exposure to airborne concentrations of such materials to no more than 1 *μ*g/m^3^. The NIOSH also recommended an occupational exposure limit for ultrafine TiO_2_ of 0.3 mg/m^3^. Among the 5 CNT and 13 TiO_2_ SDSs collected, none mentioned the OELs for CNTs or TiO_2_ (Table 7).

## 4. Discussion

While directly comparable statistics are limited on the numbers of nanomaterial handling and manufacturing workplaces and the number of potential nanomaterial-exposed workers from leading nanotechnology countries such as USA, Japan, and Germany, the numbers of workplaces and workers handling and manufacturing nanomaterials are comparable with the nanotechnology development in each country.

In Korea, the first nationwide survey on workplace environment handling and manufacturing of nanomaterials and nanoproducts was conducted in 2014, and 340 workplaces were identified as manufacturing or handling (synthesizing and processing) nanomaterials or nanoproducts, although this number increases when including workplaces that use nanomaterials for production or export. Among the 340 nanomaterial manufacturing workplaces, TiO_2_, SiO_2_, and carbon black, previously widely used before nanomaterials became an issue, are the main nanomaterials manufactured in Korea. Noticeably, new forms of nanomaterials, such as carbon nanomaterials including CNTs, CNF, and fullerene, are manufactured at more than 20 workplaces. As a result, the survey showed that 45,750 workers are potentially associated with exposure to manufactured nanomaterials, and some workplaces use more than one nanomaterial. Whenever new technology has been inappropriately introduced to the workplace with a lack of preparation for unknown hazards, Korean workers have sometimes experienced new occupational diseases. Therefore, even though the number of nanomaterial handling, processing, and manufacturing workplaces is still relatively small (0.3% of total number of workplaces) and only a few workers are exposed to nanomaterials when compared with the total number of workers, more attention is still needed for workers in areas of converging technologies and relatively new technologies, such as nanotechnology.

In terms of exposure to nanomaterials in the workplace, there are currently no practical standardized measurement and analysis methods for airborne nanomaterials in workplace air. In Korea, as regulated by the OSH Act, an employer whose workers may be exposed to hazardous materials, including nanomaterials, is responsible for measuring the work environment for hazardous materials, including nanosized particulate. The methods for measuring hazardous materials in the workplace are described in the OSH Act (notification), and MOEL-designated occupational health service institutes with special equipment and qualified personnel provide work environment measurements for the employer. Yet, nanoscale characteristics are not considered as there is no current obligation to measure nanomaterials when conducing workplace air measurements for hazardous materials. Although the workplace air measurement results in this study only revealed a few cases that exceeded or approached the OEL, some precautionary measures need to be considered to protect workers in an environment handling and manufacturing nanomaterials, especially as the OELs of nanomaterials are much lower than those of bulk materials. In particular, those workplaces exceeding the OEL should be more strictly controlled via engineering controls, PPE, and training for workers potentially exposed to nanomaterials.

As regards the special health examinations of workers potentially exposed to nanomaterials at work, 9 cases (0.73%) of suspected occupational disease among 1,230 workers are significant, requiring some form of worker protection, when compared with the proportion (1.7%) of suspected occupational disease cases in the 2014 nationwide special health examination of workers potentially exposed to typical health hazards, such as noise, organic solvents, and heavy metals. Although the proportion of suspected occupational disease for the nanomaterial workers was slightly lower than that for the workers handling and manufacturing heavy metals and organic solvents, there is still a need for special precautionary measures in workplaces handling/manufacturing nanomaterials, such as the development of biological indices related to nanomaterials, periodic examinations of workers, career records for workers handling/manufacturing nanomaterials, prevention training and sound workplace management, and accurate SDSs.

From May 1, 1995, the Korean Occupational Safety and Health Act requires the preparation of an SDS when a company transfers or supplies a chemical (except medicines and cosmetics) or chemical-containing preparation. After Korea adopted the GHS recommendation on December 12, 2006, the SDS format is now required to follow the GHS format. However, in a study by Lee et al. [23] that evaluated 97 nanomaterial-related SDSs according to the criteria set by the GHS, most of the SDSs did not include sufficient information on the safety of nanomaterials, such as their toxicity and physicochemical properties. The reasons for this lack of information in the SDS were concluded as (1) a lack of toxicity and physicochemical property information on nanomaterials, (2) unawareness of the effectiveness of conventional exposure controls, such as local exhaust ventilation and encapsulation, and personal protective equipment (PPE), in protecting against nanomaterial exposure, (3) a lack of information on emergency and firefighting measures, and (4) a lack of knowledge on how existing regulations apply to nanomaterials. Therefore, these observations led one of the coauthors to develop an international standard: ISO TR 13329 Nanomaterials: Preparation of Material Safety Data Sheet (MSDS). Hence, this paper reevaluated the preparation of nanomaterial SDSs according to ISO TR 13329 and GHS. In contrast to the study by Lee et al. [23], the present study analyzed the results of the 2014 national survey on companies handling and manufacturing nanomaterials and nanoproducts. In particular, the reliability of the collected SDSs was evaluated according to ISO TR 13339 MSDS [14] based on the inclusion of the name of a competent person(s) related to the SDS preparation, the format of the SDS, the absence of blanks under any of the headings, periodic review and reissue, the inclusion of concrete health hazard information identified in previous studies, and a nanomaterial statement in the SDS.

Unfortunately, most of the collected SDSs still did not include the characteristics and hazards of nanomaterials. Despite numerous studies and media releases on the health hazards of nanomaterials and well-known occupational exposure limits recommended by the NIOSH, such information was not stated in most of the nanomaterial SDSs. In Europe, substances including substances at the nanoscale manufactured or imported in volumes of 1 tonne or more per year must be registered under the REACH regulation [24]. In the UK, the HSE views CNTs as substances of very high concern, requiring a “precautionary approach,” such as risk management, and stated that a high level of control be used, including a recommendation to control exposure at source when carrying out all tasks, including packaging for disposal.

Although hazard information on nanomaterials is gradually becoming available to the public, the inclusion of hazard information on SDSs was found to be omitted or easily overlooked during the SDS preparation or update process. Many nanomaterial SDSs were also indistinguishable from bulk material SDSs, containing the same information. As a result, specific nanomaterial safety guidelines have been provided and recommended by international organizations such as the OECD, ISO, and WHO. Notwithstanding, tighter regulatory actions are still needed, such as the reporting rules of the EPA and Environment Canada and decree in France.

Furthermore, particular attention to the characteristics of nanomaterials and health effects is needed for nanomaterial-relevant workplaces. In addition to the precautionary measures for the health effects of bulk materials, proper precautionary measures are also needed for nanomaterials, especially since nanomaterials produce different hazards and health effects from bulk materials. Thus, as a precautionary approach for health and safety, the competent authority needs to provide specific guidelines to protect the health of workers potentially exposed to nanomaterials or nanoproducts, along with sound management of nanomaterials that considers publications by international organizations such as WHO, OECD, and ISO TC 229 and national institutes such as NIOSH. These international guidelines or standards should be introduced as legal standards in the Korean occupational safety and health framework and implemented as soon as possible. Plus, as described in ISO TR 13329, the uniqueness of nanomaterials should be incorporated in the current SDS preparation guidelines.

When taken together, the current analysis of the first national survey of workplaces handling and manufacturing nanomaterials, the exposure to and health effects of nanomaterials, and the evaluation of nanomaterial safety data sheets all provide valuable national-level information on the exposure and health status of workers relevant to nanomaterials. While only a few cases of suspected occupational diseases were identified and worker exposure to particulate material was lower than current OELs, precautionary measures should still be taken to reduce exposure to nanomaterials, as well as other hazardous materials, to protect workers from health hazards.

## Supplementary Material

Supplemental Table 1. The amount of nanomaterials used.

## Figures and Tables

**Table 1:** Total number of workplaces surveyed.

Classification	Number (%)
Total number of workplaces surveyed	126,846
Level of workplace	
Workplaces hiring 5 workers or more, insured by OACI in manufacturing sector	100,773 (79.4%)
Workplaces hiring fewer than 5 workers, insured by OACI in manufacturing sector	16,073 (12.7%)
Workplaces in nonmanufacturing sector	10,000 (7.9%)
Total number of workplaces handling or manufacturing nanomaterials	340 (0.27%) (100%)
Types of handling or manufacturing	
Synthesis	75 (22.1%)
Processing	121 (35.5%)
Production	75 (22.1%)
Other	69 (20.3%)
Number of nanomaterials or products	
Number of nanomaterials	546
Number of nanoproducts	583
Average number of nanomaterials per company	1.60
Average number of nanoproducts per company	1.71

**Table 2:** Number of nanomaterials handled or manufactured or manufactured for product.

Total	TiO_2_	SiO_2_	Carbon Black	Ag	Al_2_O_3_	ZnO	Pb	CeO_2_	Polymers	Fe	Au	CNTs & CNFs	Other
583	91	88	84	35	35	34	33	31	23	23	21	20	65

**Table 3:** Results of workplace air measurements (unit: numeric, %).

	Total	TiO_2_	SiO_2_	Al_2_O_3_	Ag	ZnO	Fe	Others
Number of companies: conducted workplace air measurements	136 (100)	40 (100)	32 (100)	17 (100)	13 (100)	13 (100)	6 (100)	15 (100)
Number of companies: nondetected	34 (25.0)	17 (42.5)	4 (12.5)	2 (11.8)	5 (38.5)	1 (7.7)	2 (33.3)	3 (20.0)
Number of companies: detected	29 (21.3)	1 (2.5)	8 (25.0)	7 (41.2)	0 (0.0)	9 (69.2)	2 (33.3)	2 (13.3)
Lowest level (mg/m^3^)	—	0.0001	0.009	0.005		0.001	0.008	0.013
Highest level (mg/m^3^)	—	0.001	3.72	9.17		0.70	1.16	0.02
OEL (mg/m^3^)	—	10	10^a^ 0.05^b^	10	0.1	2^c^	1^d^	—
Number of companies: no data for materials	73 (53.7)	22 (55.0)	20 (62.5)	8 (47.0)	8 (61.5)	3 (23.1)	2 (33.3)	10 (66.7)

^a^Diatomaceous earth; ^b^silica, crystalline quartz; ^c^respirable fraction; ^d^iron salts; OEL: occupational exposure limit.

**Table 4:** The results of health examination.

	Total	SiO_2_	ZnO	Others^*∗*^
Total number of workplace: health examination implemented	68 (100)	32 (100)	5 (100)	31 (100)
Number of companies: health examination for the hazardous materials implemented	48 (70.6)	16 (50.0)	5 (100)	27 (87.1)
Number of workers whose health status examined	1,230	385	44	801
Number of workers: occupational disease suspected	9	2	2	5
Expected target organ		Not recorded	Respiratory system	Circulatory system (1)
Number of workers requiring follow-up health-exam regularly	7	5	1	1
Expected target organ		Respiratory system (3) Not recorded (2)	Respiratory system	
Number of companies: implemented health examination, but no workers examined for the hazardous materials	20 (29.4)	16 (50.0)	0 (0.0)	4 (12.9)

^*∗*^Others: CoO, Pb, SnO_2_, Al_2_O_3_, Fe, copper, nickel, manganese, antimony, vanadium pentoxide, zirconium, cadmium, chromium, arsenic trioxide, and so forth.

**Table 5:** Collection of nanomaterial SDSs from various manufacturing industries.

Type of manufacturing industry	Number of workplaces	Number of SDSs
Electronic components or semiconductor devices	9	56
Organic chemical products	5	17
Rubber products	4	13
Inorganic chemical products	4	8
Synthesis of resins	2	5
Paint or similar products	2	3
Other	5	23

Total	31	125

**Table 6:** Number of SDSs collected by nanomaterial or nanoproduct

Nanomaterial	Number of SDSs
Total	125 (100%)
CeO_2_	25 (20.0%)
SiO_2_	21 (16.8%)
Carbon black	15 (12.0%)
TiO_2_	13 (10.4%)
Al_2_O_3_	8 (6.4%)
Ag	6 (4.8%)
CNTs	5 (4.0%)
Other	32 (25.6%)

**Table 7:** SDS content evaluation.

	Total number of SDSs	Yes	No
Written in Korean	125 (100%)	110 (88.0%)	15 (12.0%)
Information on competent person	125 (100%)	19 (15.2%)	106 (84.8%)
Description of recommended use	125 (100%)	98 (78.4%)	27 (21.6%)
Prepared according to OSH Act or GHS	125 (100%)	111 (88.8%)	14 (11.2%)
Relevant information under all headings, including “not available”	125 (100%)	108 (86.4%)	17 (13.6%)
Updated within 3 years	125 (100%)	51 (40.8%) 26 (20.8%) still within 3 years of being issued	48 (38.4%)
Health hazard information description	85 (100%)	21 (24.7%)	64 (75.3%)
Hazard identification of nanomaterials	85 (100%)	15 (17.6%)	70 (82.4%)
Statement of manufactured nanomaterial	125 (100%)	4 (3.2%)	121 (96.8%)
Stated cleanup method for spills, leaks, or releases	125 (100%)	3 (2.4%)	122 (97.6%)
Stated cleanup method for dry manufactured nanomaterials	125 (100%)	4 (3.2%)	121 (96.8%)
Stated wet-wiping method for liquid spills	125 (100%)	3 (2.4%)	122 (97.6%)
OEL for CNTs	5 (100%)	0 (0%)	5 (100%)
OEL for TiO_2_	13 (100%)	0 (0%)	13 (100%)
