# Supplementary material for: National Survey of Workplaces Handling and Manufacturing Nanomaterials, Exposure to and Health Effects of Nanomaterials, and Evaluation of Nanomaterial Safety Data Sheets
Source: Biomed Res Int. 2016 Jul 31;2016:8389129. doi: 10.1155/2016/8389129 (PMC4983336; doi:10.1155/2016/8389129)
Supplement: Supplementary file 1 — Supplemental Table 1. The amount of nanomaterials used. [file 8389129.f1.docx]

Supplemental Table 1. The amount of nanomaterials used

| Ranking | Nanomaterials | Amount (Ton/Year) | Use |
| --- | --- | --- | --- |
|  | Total | 8,704.6 |  |
| 1 | SiO_2_ | 4,779.6 | Packaging, cosmetics, etc |
| 2 | TiO_2_ | 3,902.2 | Paints, construction materials, coatings, cosmetics, etc |
| 3 | ZnO | 12.5 | Fabrics, home appliances, paints, inks, etc |
| 4 | CNT | 3.0 | Fabrics, electrical appliances, household items, sporting goods, etc |
| 5 | Ag | 0.3 | Packaging, fabrics, household items, sporting goods, paints, inks, etc |

<Source, A research on nanomaterial inventory establishment scheme, Ministry of Environment, 2010>

NSTC (National Science and Technology Commission) (2011), The First Nanosafety Management Master Plan (2012-2016), National Science and Technology Commission, Seoul, Korea (in Korean), http://www.nnpc.re.kr/knowledge/nano_policy/19/view?p_page=1&p_pagesize=10&o_field=&o_direction=&s_type=&s_keyword=&s_category= accessed 1/23/2016
